# Incidence and risk factors of new clinical disorders in patients with COVID-19 hyperinflammatory syndrome

**DOI:** 10.1038/s41598-025-04070-9

**Published:** 2025-06-06

**Authors:** Kevin E. Duong, Justin Y. Lu, Stephen Wang, Tim Q. Duong

**Affiliations:** 1 Department of Radiology, Albert Einstein College of Medicine and Montefiore Medical Center, Bronx, NY USA; 2 Department of Surgery, Beth Israel Deaconess Medical Center and Harvard Medical School, Boston, MA USA; 3 Center for Health & Data Innovation, Albert Einstein College of Medicine and Montefiore Medical Center, Bronx, NY USA

**Keywords:** Long COVID, Post-acute sequela of SARS-CoV-2 infection (PASC), Cytokine storm, Medical research, Rheumatology, Infectious diseases

## Abstract

This study investigated new incident clinical disorders in patients with COVID-19-related hyperinflammatory syndrome (cHIS) 3.5 years post infection. We analyzed 14,335 patients hospitalized with COVID-19 from March-2020 to July-2023. cHIS was defined based on a point system that included elevated body temperature, macrophage activation, hematological dysfunction, coagulopathy, and hepatic enzyme. Outcomes were newly diagnosed disorders of hypertension, diabetes, cardiovascular diseases, chronic kidney disease (CKD), chronic obstructive pulmonary disease (COPD), and asthma post COVID-19. Cumulative incidences and hazard ratios were computed. Compared to non-cHIS patients, cHIS patients were older, fewer female, more Blacks, higher prevalence of pre-existing comorbidities. Patients with cHIS had higher risk of developing cardiovascular disease (HR = 1.24 [1.04,1.47] *p* < 0.05), CKD (1.24 [1.01, 1.53] *p* < 0.05), and obesity (1.61 [1.31,1.98], *p* < 0.001) but not hypertension, diabetes, COPD, and asthma. Cumulative incidence analysis showed that patients ≥ 50 years old showed markedly higher new incidences of individual new disorders compared to patients < 50 years old. COVID-19 related hyperinflammatory syndrome confers a significantly higher risk for developing new common clinical disorders. Identifying risks for developing new clinical disorders in patients with COVID-19 related hyperinflammatory syndrome may encourage diligent follow-up of high-risk individuals.

**Supplementary Information:**

The online version contains supplementary material available at 10.1038/s41598-025-04070-9.

## Introduction

The coronavirus disease 2019 (COVID-19) pandemic, caused by severe acute respiratory syndrome coronavirus 2 (SARS-CoV-2)^1,2^, has posed an unprecedented global health challenge. Although SARS-CoV-2 infection is primarily recognized for its respiratory manifestations, a growing body of evidence suggests that severe COVID-19 is accompanied by a pathological hyperinflammatory response, the consequences of which extend beyond pulmonary dysfunction^3,4^. The pathophysiology of severe COVID-19 encompasses immune responses that include hyperactivation of immune cells and a disproportionate release of pathologic inflammatory cytokines. This phenomenon is reminiscent of a cytokine storm and has garnered significant attention as a potential driver of the clinical severity and complications observed in severe COVID-19. The pathologic hyperinflammatory responses in COVID-19 include an uncontrolled release of interleukins (IL), tumor necrosis factor (TNF), C-reactive protein, D-dimer, ferritin, neutrophils and lymphocytes, and other inflammatory modulators, resulting in a systemic cascade of immune dysregulation.

Although incompletely characterized, the COVID-19 related hyperinflammatory syndrome (cHIS) shares similarities with other hyperinflammatory disorders^5–7^. A few studies have shown that cHIS is correlated with worse acute COVID-19 outcomes and cHIS is the target for immunomodulatory therapeutics^3–7^. However, it is unclear whether patients with cHIS during acute COVID-19 exhibit worse long-term outcomes.

It is possible that the acute insults of pathologic inflammatory syndromes associated with SARS-CoV-2 infection could increase long-term susceptibility to developing new clinical disorders among COVID-19 survivors^8,9^. These include increased risk of developing chronic diseases such as hypertension, diabetes, cardiovascular disease (CVD), chronic kidney disease (CKD), COPD, and asthma due to persistent systemic inflammation, endothelial dysfunction, immune dysregulation, and tissue damage^5–7^. Pro-inflammatory cytokines like IL-6, TNF-α, and IL-1β contribute to endothelial damage and renin-angiotensin system (RAS) dysregulation, leading to long-term vascular dysfunction and an increased risk of hypertension. Similarly, cytokine-driven pancreatic β-cell injury and insulin resistance heighten susceptibility to new-onset diabetes or worsening preexisting conditions. Cardiovascular complications arise due to persistent vascular inflammation, hypercoagulability, myocardial fibrosis, and autonomic dysregulation, increasing the likelihood of myocardial infarction, stroke, and heart failure. In the kidneys, inflammatory and thrombotic processes contribute to acute kidney injury, which, when unresolved, progresses to CKD. The lungs are also affected, as chronic inflammation, oxidative stress, and airway remodeling promote the onset or acceleration of COPD and asthma, with lingering immune dysregulation exacerbating airway hyperresponsiveness. Moreover, the long-term impact of these inflammatory processes is compounded by the persistence of immune activation, epigenetic modifications, and impaired organ function, leaving survivors of severe COVID-19 at an elevated risk of developing or worsening these chronic conditions. Given these long-term risks and the sheer number of the individuals affected by COVID-19, it is important to identify early on which COVID-19 patients are at risk of developing new-onset disorders and identify risk factors to enable more diligent follow-up care to prevent long-term complications and clinical disorders.

The goals of this study were to investigate the incidence, characteristics, and risk factors of newly diagnosed clinical disorders in patients with COVID-19-related hyperinflammatory syndrome 3.5 years post infection. These new disorders included hypertension, diabetes, cardiovascular diseases, chronic kidney disease, COPD and asthma. Comparisons were made with COVID-19 patients with and without cHIS who were hospitalized for COVID-19. Predictive models were used to identify risk factors associated with new clinical disorders.

## Methods

### Study design

This retrospective study was approved by the Einstein-Montefiore Institutional Review Board (#2020-11389) with an exemption for informed consent and a HIPAA waiver and was performed in accordance with relevant guidelines and regulations. The Montefiore Health System is the largest healthcare system in the Bronx and its environs with multiple hospitals and outpatient clinics, serving a large, low-income, and racially and ethnically diverse population that was hit hard by COVID-19 early in the pandemic and subsequent surges of infections^10^. Deidentified EMR data were in the OMOP Common Data Model version 6. OMOP stores the health data, which comes from many sources, into standard vocabulary concepts. This facilitates the systematic analysis of different observational databases, which includes data from the electronic medical record system, disease classification systems and administrative claims such as SNOWMED, ICD-10, LOINC, etc. DB Browser for SQLite (version 3.12.0) was used to export and query data as SQLite database files. We relied on the accuracy of EMR and there could be errors in documentation as in any large database. Over the years, we have conducted chart reviews on a subset of patients and found that all automated extracted variables, which clinicians rely on for patient care, were accurate and consistent with clinical notes. Studies using earlier versions of this large database to address different questions have been published previously^11–19^.

Only hospitalized patients were studied because they had blood markers needed to define cHIS during acute COVID-19. Data were obtained from March 11, 2020 to July 1, 2023. COVID-19 positive patients were defined by PCR test. For new onset disorders, patients who did not have at least one inpatient, outpatient or ER visit > 30 days after the index date (PCR positive test) were excluded from final analysis. Patients with a specific pre-existing disorder were excluded for that specific disorder, as the aim of the study was to identify patients with new-onset disorders. Observational Medical Outcomes Partnership (OMOP) concept id’s of the common clinical disorders are summarized in **Supplemental Table 1**.

### Data extraction

Demographic data (e.g., age, sex, ethnicity, race), temperature, pre-existing comorbidities (hypertension, diabetes, cardiovascular diseases, chronic kidney disease (CKD), diabetes, chronic obstructive pulmonary disease (COPD), asthma, and obesity), and laboratory tests (lymphocyte count, hemoglobin, platelet count, D-dimer, lactate dehydrogenase (LDH), aspartate aminotransferase (AST), triglyceride, C-reactive protein, white blood cell count (WBC), and creatinine. Invasive mechanical ventilation (IMV) and ICU admission status were also extracted. COVID-19 hospitalization status was also tabulated. These data were the average of multiple measurements obtained during acute infection within two weeks of positive COVID-19 PCR test.

### Definition of cHIS

 cHIS was defined based on a point system^4^ in which one point is given for each of the six criteria: fever (temperature >38 °C), macrophage activation (ferritin >700 µg/L), hematological dysfunction (neutrophil: lymphocyte >10 or both hemoglobin<9.2 g/dL and platelet count <110 × 10^9^ cell/L), coagulopathy (D-dimer>1.5 µg/L), hepatic injury (LDH> 400U/L) or AST>100U/L, cytokinaemia (IL-6>15pg/mL, triglyceride>150 mg/dL, or CRP>15 mg/dL). A patient with a cHIS score of >=3 was considered to have cHIS and <3 was considered no cHIS. Each variable used to calculate cHIS score was the average of multiple measurements obtained during acute infection within two weeks of positive COVID-19 PCR test.

### Primary outcomes

The primary outcome was the development of new-onset hypertension, diabetes, cardiovascular diseases (defined as a composite of coronary artery disease, chronic heart failure and myocardial infarction), chronic kidney disease, COPD, and asthma at least one month post COVID-19.

### Statistical analysis

Group differences in disease frequency and percentages for categorical variables were tested using the χ^2^ test. Group comparison for categorical variables used χ^2^ exact tests, and for continuous variables, the Mann-Whitney test was used. Cumulative incidence function for each new-onset disorder was calculated. Univariate and multivariable Cox proportional hazard models were used to estimate hazard ratios (HR) and 95% CI, adjusting for demographics and comorbidities. Patients were censored at death or their last follow up within the health system. P-values <0.05 were considered statistically significant.

## Results

**Fig. 1:**
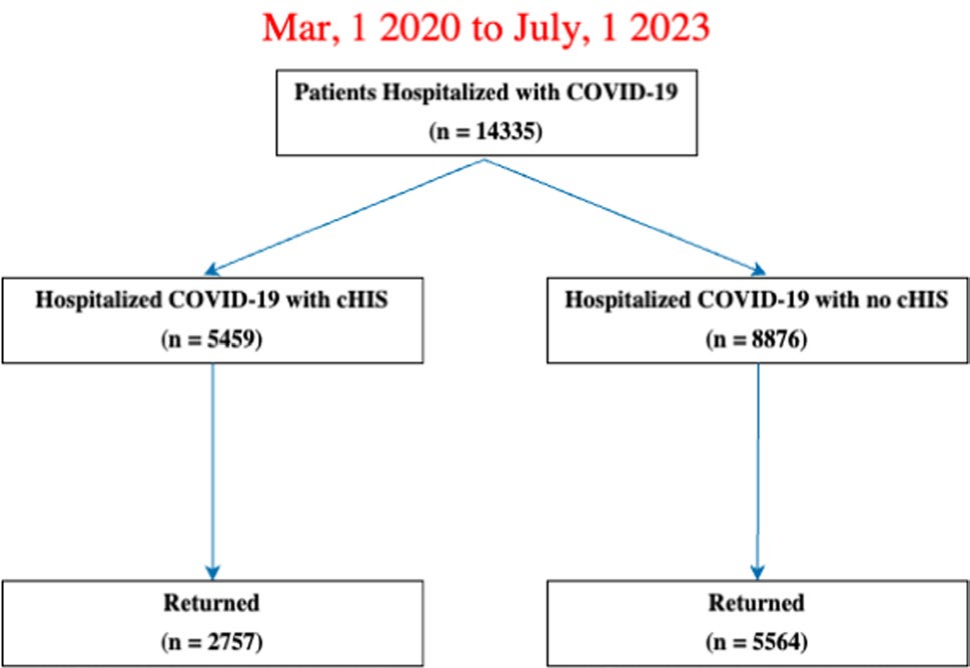
Flowchart for patient selection of COVID-19 hospitalized patients with cHIS, COVID-19 hospitalized patients with no cHIS, and non-hospitalized COVID-19 patients from Mar 1, 2023 to Jan 1, 2023.

Figure 1 describes the flowchart for patient selection. From March 2020 to July 2023, there were 14,355 hospitalized COVID-19 patients, of which 5,459 had cHIS and 2,757 returned to our health system at least 1 month post COVID-19, and 8,876 had no cHIS and 5,564 returned. Patients who returned were younger, more female, and had higher prevalence of hypertension, diabetes, COPD, asthma, cardiovascular disease, CKD and obesity compared to those that didn’t return due to large sample sizes (Supplemental Table 2). These differences are not clinically significant.

Table 1 summarizes the demographics, comorbidities and new-onset conditions for patients who survived and returned to the health system, grouped by cHIS status. The cHIS cohort was older (p < 0.001) and consisted of more Blacks (p < 0.001) and fewer female (p < 0.001) compared to no cHIS patients. The cHIS cohort had a higher prevalence of pre-existing hypertension, diabetes, cardiovascular diseases, CKD, COPD (p < 0.05), and lower prevalence of asthma (p > 0.05). More cHIS patients were treated with ICU/IMV (16.50% vs 4.15%, p < 0.001) compared with non-cHIS patients during COVID-19 hospitalization.

**Table 1:** Characteristics of cHIS and no cHIS COVID-19 hospitalized patients who returned and survived 30 days post COVID to the health system.

	cHIS (*n* = 2757, 33.1%)	No cHIS (*n* = 5564, 66.9%)
Demographics		
Age, median (IQR)	69 (21) ***	63 (35)
Female %	1267 (45.96%) ***	3376 (60.68%)
White	296 (10.74%)	630 (11.32%)
Black	1093 (39.64%) ***	1917 (34.45%)
Asian	87 (3.16%)	175 (3.15%)
Other	1281 (46.5%) ***	2842 (51.1%)
Hispanic	1132 (41.06%) ***	2630 (47.27%)
Comorbidities		
Hypertension	1961 (71.13%) ***	3507 (63.03%)
Diabetes	1502 (54.48%) ***	2424 (43.57%)
COPD	407 (14.76%) *	937 (16.84%)
Asthma	604 (21.91%) ***	1647 (29.60%)
Cardiovascular Disease	1111 (40.30%) ***	2032 (36.52%)
CKD	1091 (39.57%) ***	1594 (28.65%)
Obesity or BMI > 30	1312 (47.59%)	2583 (46.42%)
Smoking	994 (36.05%)	2011 (36.14%)
Lab Values at index date, mean (sd)		
Temperature (°F)	100.93 (1.46) ***	99.58 (1.87)
Ferritin (µg/L)	1588.15 (1590.48) ***	374.55 (572.47)
Neutrophil (k/uL)	10.33 (7.94) ***	6.76 (4.61)
Lymphocytes (k/uL)	1.88 (3.39) **	2.08 (2.23)
Hemoglobin (g/dL)	12.89 (2.20) ***	12.66 (2.01)
Platelets (k/uL)	345.74 (143.13) ***	283.13 (119.42)
D-dimer (mg/L)	5.22 (5.91) ***	1.60 (2.71)
LDH (U/L)	530.80 (421.65) ***	275.14 (124.89)
AST (U/L)	103.38 (317.57) ***	43.34 (97.41)
Triglyceride (mg/dL)	183.83 (191.42) ***	116.34 (74.26)
CRP (mg/L)	152.81 (104.47) ***	50.54 (59.79)
WBC (k/uL)	12.95 (10.96) ***	9.37 (7.26)
Creatinine (mg/dL)	2.73 (3.59) ***	1.59 (2.16)
COVID-19 ICU/IMV	455 (16.50%) ***	231 (4.15%)
New-onset conditions (%)		
Hypertension	186/796 (23.37%) ***	250/2057 (12.15%)
Diabetes	103/1255 (8.21%) *	194/3140 (6.18%)
COPD	113/2350 (4.81%) *	166/4627 (3.59%)
Asthma	70/2153 (3.25%)	141/3917 (3.60%)
Cardiovasc Disease	249/1646 (15.13%) ***	313/3532 (8.86%)
CKD	156/1666 (9.36%) ***	231/3970 (5.82%)
Obesity	167/1445 (11.56%) ***	235/2981 (7.88%)

*Indicates *p* < 0.05, ** *p* < 0.01, *** *p* < 0.001 between cHIS and no cHIS patients. Lab values are the average readings within two weeks of index date.

With respective to outcomes, cHIS patients had higher new incident hypertension (23.37% vs. 12.15%, *p* < 0.001), diabetes (8.21% vs. 6.18%, *p* < 0.05), COPD (4.81% vs. 3.59%, *p* < 0.05), cardiovascular diseases (15.13% vs. 8.86%, *p* < 0.001), CKD (9.36% vs. 5.82%, *p* < 0.001), and obesity (11.56% vs. 7.88%, *p* < 0.001).

Patient profiles for all hospitalized patients, stratified by cHIS status are shown in Supplemental Table 3. Overall, the profiles of cHIS and no cHIS cohorts were similar to the final cohort analyzed. Of note, cHIS patients had higher proportions of ICU/IMV (23.59% vs. 5.03%, *p* < 0.001), in-hospital mortality (22.48% vs. 2.68%, *p* < 0.001) and mortality post COVID-19 (at least after 30 days) (23.61% vs. 3.1%, *p* < 0.001).

**Fig. 2:**
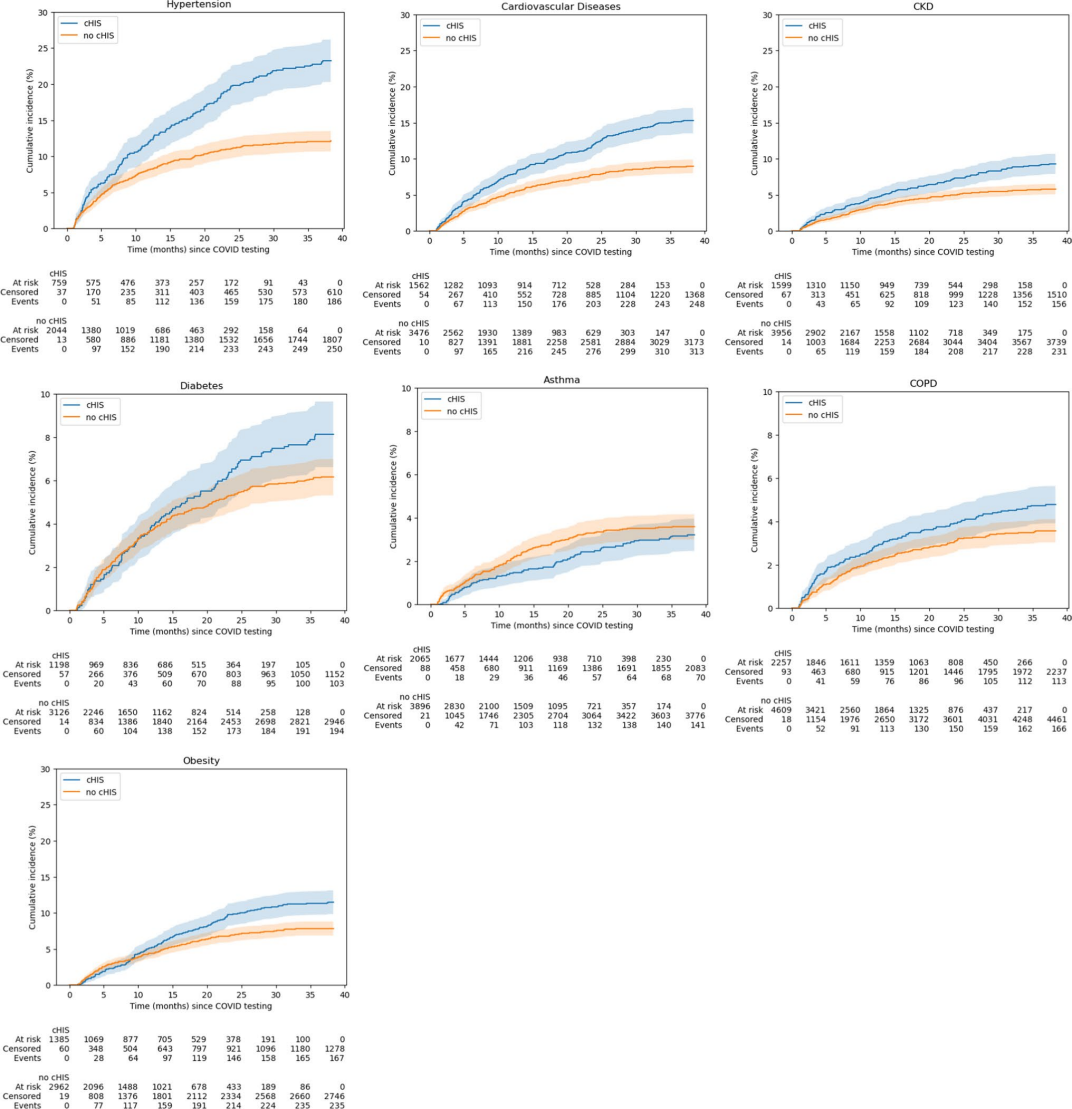
Cumulative incidence of new hypertension, diabetes, cardiovascular diseases, chronic kidney disease, COPD and asthma.

The cumulative incidences for developing new disorders for cHIS and no cHIS patients are plotted in Fig. 2. Overall, cHIS patients had a higher new incident hypertension, cardiovascular disease, CKD, diabetes, COPD and obesity than no cHIS patients (*p* < 0.05). The adjusted hazard ratios for cHIS status for developing new disorders were computed (Table 2) with sex, ethnicity, race, hypertension, diabetes, COPD, asthma, cardiovascular disease, CKD, smoking and obesity as covariates. cHIS status was associated with higher significant hazard ratio of new incident cardiovascular disease (HR = 1.24 [1.04,1.47] *p* < 0.05), CKD (1.24 [1.01, 1.53] *p* < 0.05), and obesity (1.61 [1.31,1.98], *p* < 0.001) but not hypertension, diabetes, COPD, and asthma. For reference, univariable HR results are shown in Supplemental Table 4.

**Table 2:** Multivariable hazard ratios for developing new hypertension, diabetes, cardiovascular diseases, chronic kidney disease, COPD and asthma.

	Hypertension	Diabetes	COPD	Asthma	Cardiovascular disease	CKD	Obesity
cHIS	1.20 (0.98, 1.47)	1.08 (0.85, 1.38)	1.13 (0.89, 1.44)	1.11 (0.82, 1.49)	1.24 (1.04, 1.47)*	1.24 (1.01, 1.53)*	1.61 (1.31, 1.98)***
Gender	0.82 (0.67, 1.00)	0.89 (0.69, 1.13)	0.83 (0.64, 1.06)	1.30 (0.98, 1.73)	0.78 (0.66, 0.93)*	0.73 (0.59, 0.90)***	1.36 (1.11, 1.67)***
Age	1.02 (1.02, 1.03)***	1.01 (1.01, 1.02)***	1.03 (1.03, 1.04)***	0.97 (0.97, 0.98)***	1.03 (1.02, 1.03)***	1.03 (1.02, 1.04)***	1.00 (0.99, 1.00)
Hispanic	1.17 (0.92, 1.50)	1.13 (0.84, 1.51)	0.81 (0.60, 1.11)	1.18 (0.83, 1.68)	0.97 (0.78, 1.21)	1.20 (0.92, 1.57)	1.35 (1.05, 1.73)*
Black	1.24 (0.96, 1.60)	1.13 (0.83, 1.52)	1.06 (0.78, 1.46)	1.23 (0.86, 1.76)	1.01 (0.81, 1.26)	2.01 (1.54, 2.62)***	1.04 (0.80, 1.36)
Hypertension	N/A	1.48 (1.11, 1.97)*	1.38 (0.98, 1.93)	1.23 (0.86, 1.75)	1.47 (1.18, 1.83)***	1.63 (1.20, 2.22)***	0.98 (0.76, 1.25)
Diabetes	1.41 (1.13, 1.76)***	N/A	1.11 (0.86, 1.44)	0.89 (0.65, 1.23)	1.28 (1.07, 1.53)*	1.60 (1.30, 1.98)***	1.40 (1.11, 1.75)***
COPD	0.83 (0.59, 1.16)	0.86 (0.61, 1.21)	N/A	2.95 (2.07, 4.2)***	1.36 (1.06, 1.74)*	1.12 (0.85, 1.48)	0.82 (0.59, 1.14)
Asthma	0.94 (0.73, 1.20)	1.26 (0.96, 1.64)	2.41 (1.85, 3.14)***	N/A	1.18 (0.97, 1.45)	1.05 (0.82, 1.34)	0.93 (0.72, 1.21)
Cardiovascular disease	1.18 (0.93, 1.50)	1.69 (1.30, 2.20)***	1.35 (1.05, 1.74)*	1.68 (1.20, 2.36)***	N/A	1.41 (1.14, 1.74)***	0.84 (0.66, 1.07)
CKD	1.33 (1.03, 1.72)*	1.20 (0.91, 1.57)	1.07 (0.83, 1.39)	0.99 (0.71, 1.39)	1.32 (1.1, 1.59)***	N/A	0.77 (0.60, 0.98)*
Smoking	1.02 (0.82, 1.26)	0.88 (0.68, 1.14)	1.77 (1.39, 2.25)***	1.43 (1.05, 1.94)*	0.93 (0.78, 1.12)	0.81 (0.65, 1.02)	1.02 (0.81, 1.28)
Obesity	1.36 (1.12, 1.65)***	1.47 (1.16, 1.86)***	1.19 (0.93, 1.52)	1.00 (0.76, 1.32)	1.27 (1.07, 1.51)*	1.19 (0.97, 1.47)	N/A

*Indicates *p* < 0.05, ^**^ *p* < 0.01, ^***^ *p* < 0.001.

To explore the effect of age, new incident disorders for cHIS and no cHIS patients stratified by age < 50 and ≥ 50 years old (Table 3). Patients ≥ 50 years old showed higher incidence of individual new disorders compared to patients < 50 years old. The effects were most dramatic with all new disorders except asthma which showed less age-related effects.

**Table 3:** New incident disorders for cHIS and no cHIS patients stratified by age < 50 and ≥ 50 years old.

	cHIS (2757)		No cHIS (5564)	
	Age > = 50: (*n* = 2365)	Age < 50: (*n* = 392)	Age > = 50: (*n* = 3811)	Age < 50: (*n* = 1753)
Hypertension	156/554 (28.16%) ^^^	30/242 (12.40%)	154/692 (22.25%) ^^^	96/1365 (7.03%)
Diabetes	87/983 (8.85%)	16/272 (5.88%)	154/1655 (9.31%) ^^^	40/1485 (2.69%)
COPD	111/1974 (5.62%) ^^^	2/376 (0.53%)	153/2937 (5.21%) ^^^	13/1690 (0.77%)
Asthma	55/1862 (2.95%) ^	15/291 (5.15%)	90/2709 (3.32%)	51/1208 (4.22%)
Cardiovascular Disease	220/1322 (16.64%) ^^^	29/324 (8.95%)	281/1889 (14.88%) ^^^	32/1643 (1.95%)
CKD	149/1359 (10.96%) ^^^	7/307 (2.28%)	206/2357 (8.74%) ^^^	25/1613 (1.55%)
Obesity	137/1265 (10.83%) ^	30/180 (16.67%)	160/2054 (7.79%) ^^^	75/927 (8.09%)
Hypertension	156/554 (28.16%) ^^^	30/242 (12.40%)	154/692 (22.25%) ^^^	96/1365 (7.03%)
Diabetes	87/983 (8.85%)	16/272 (5.88%)	154/1655 (9.31%) ^^^	40/1485 (2.69%)

To confirm whether cHIS was valid predictor, we also evaluated whether cHIS is associated with acute COVID-19 severity (*acute* COVID-19 critical illness and in-hospital mortality). With the cHIS threshold >3, cHIS predicted critical illness and mortality with an AUC of 0.84, and 0.75, respectively. With the cHIS >2, cHIS predicted critical illness and mortality with an AUC of 0.74 and 0.69, respectively. These AUC, sensitivity and specificity values are summarized in **Supplemental Table 5**. Note that sensitivity was higher than specificity as expected due to asymmetry of outcomes.

## Discussion

This study investigated the association of cHIS and new clinical disorders following SARS-CoV-2 infection and identified risk factors associated for developing these new clinical disorders. The major findings are: (i) Compared to non-cHIS patients, cHIS patients were older, had fewer female, more Blacks, higher prevalence of pre-existing comorbidities, (ii) cHIS status was significantly associated with the development of new cardiovascular disease (HR = 1.24 [1.04,1.47] *p* < 0.05), chronic kidney disease (1.24 [1.01, 1.53] *p* < 0.05), and obesity (1.61 [1.31,1.98], *p* < 0.001) but not hypertension, diabetes, COPD, and asthma, (iii) patients ≥ 50 years old show higher incidence of new individual disorders compared to patients < 50 years old.

Manson et al. assessed the outcomes of hyperinflammation in 269 COVID-19 patients admitted to their UK hospital system in March 2020^20^. Hyperinflammation was defined as a C-reactive protein concentration greater than 150 mg/L or doubling within 24 h from greater than 50 mg/L, or a ferritin concentration greater than 1500 µg/L. They found patients with hyperinflammation were significantly associated with the risk of next-day escalation of respiratory support or death.

Webb et al. established the cHIS criteria to develop a scoring system that would accurately predict in-hospital mortality and initiation of invasive mechanical ventilation^4^. In their cohort of 299 patients primarily from the first wave of COVID pandemic, they found that cHIS patients with > 2 score threshold were older, more male, and had more diabetes, hypertension, arrhythmias, congestive heart failure, liver disease, obesity and had more elevated lab profiles on admission. Furthermore, they found that their cHIS threshold > 3 predicted in-hospital mortality and initiation of invasive mechanical ventilation with AUC of 0.92 and 0.81 respectively. Our results provided further support of the validity of cHIS score as a good predictor of worse acute outcomes in a more diverse population, with large proportion of Black and Hispanic.

In addition, we also applied the cHIS score to investigate the long-term outcomes of COVID-19 patients who experienced hyperinflammation during acute COVID-19 illness. Our study provided alarming statistics that hyperinflammation during acute COVID-19 illness were at higher risks for new incident hypertension, cardiovascular disease, kidney disease, and obesity at the population. Pro-inflammatory cytokines like TNF-α and IL-6 can damage the vascular endothelium, impairing its ability to produce nitric oxide, a crucial vasodilator, thereby increasing vascular resistance and blood pressure^21^. Cytokine storms could enhance the production of reactive oxygen species (ROS), leading to oxidative stress that damages vascular tissues and contributes to hypertension^22^. Inflammatory cytokines could also activate the sympathetic nervous system, resulting in increased heart rate and blood pressure. They could also stimulate the renin-angiotensin-aldosterone system (RAAS), causing vasoconstriction and sodium retention, which further elevates blood pressure.

The chronic inflammation plays a significant role in the development of new cardiovascular diseases. Persistent inflammation promotes the formation of atherosclerotic plaques by recruiting inflammatory cells such as macrophages to the blood vessel walls, leading to plaque buildup and cardiovascular events^23^. Moreover, cytokine storms can cause direct myocardial damage, resulting in myocarditis and heart failure. Inflammation also increases the risk of thrombosis by altering the coagulation pathway, promoting the formation of blood clots that can cause heart attacks and strokes^24^. Lastly, prolonged inflammation can lead to adverse cardiac remodeling, affecting heart function and contributing to heart failure^25^.

Inflammatory cytokines can damage the glomeruli, the kidney’s filtering units, leading to impaired kidney function^26^. Persistent inflammation also promotes interstitial fibrosis, or scarring, of kidney tissue, which progressively reduces kidney function over time^27^. Additionally, endothelial dysfunction within the kidneys, similar to that seen in blood vessels, impairs blood flow and filtration efficiency. The inflammation-induced damage to the glomeruli can cause protein leakage into the urine, known as proteinuria, which is a key indicator of CKD progression^26^.

Obesity is also closely linked to chronic low-grade inflammation, where adipose tissue secretes pro-inflammatory cytokines that contribute to systemic inflammation^28^. These inflammatory cytokines interfere with insulin signaling pathways, leading to insulin resistance, a critical factor in the development of obesity and type 2 diabetes. Inflammation also disrupts normal metabolic processes, resulting in dysregulation of appetite, energy expenditure, and fat storage^29^. Moreover, inflammation can cause the release of free fatty acids from adipose tissue, leading to lipotoxicity, which is harmful to other organs such as the liver and pancreas.

To our knowledge, this is the first study that reported the long-term effects of acute COVD-19 related hyperinflammation on new incident major clinical disorders.

### Confounders

Confounders such as hypertension, diabetes, COPD, asthma, CVD, CKD, smoking, and obesity play a crucial role in influencing outcomes and they are interrelated^5–7^. These conditions often contribute to systemic inflammation, metabolic dysregulation, and vascular dysfunction, which can predispose individuals to developing new clinical disorders. Hypertension and cardiovascular disease are closely linked, as chronic high blood pressure leads to endothelial damage, increased arterial stiffness, and heightened cardiovascular risk. Similarly, preexisting hypertension exacerbates kidney damage by impairing renal perfusion and accelerating CKD progression. Diabetes is a well-established risk factor for multiple organ dysfunctions, including cardiovascular and renal complications, due to chronic hyperglycemia-induced endothelial damage and inflammation. COPD and asthma, while primarily respiratory conditions, are associated with systemic inflammation and oxidative stress, potentially influencing cardiovascular and metabolic outcomes. Smoking and obesity are both major contributors to chronic disease progression. Smoking induces oxidative stress, promotes vascular damage, and increases the risk of CVD, CKD, and hypertension. Obesity is a key driver of metabolic syndrome and systemic inflammation, increasing susceptibility to hypertension, CVD, and CKD. Age is also major confounder. Age also interacts with other confounders. Our outcomes were adjusted for these confounders.

### Limitations

Our study has several limitations. These findings were restricted to patients who returned to our health system. It is also possible that patients who returned were more likely to have more severe COVID-19. Montefiore is the predominant health system in the Bronx and its environs. Most patients had prior interactions with our health system before COVID-19 and continued to seek care within our system for various medical reasons over the study period. Patient data obtained via electronic medical records only included those who returned for any medical reason, including but not limited to routine office visits. It is also possible that some patients had previously undiagnosed conditions, which could result in misclassification of disorders as new onsets. However, this misclassification likely occurred to similar extents in both groups and should not alter our conclusions. Incidence of new disorders across the pandemic might be affected by other factors, including the vaccination rate, strain of SARS-CoV-2, testing rate, naturally acquired immunity, treatments, population profile, and disease severity. Vaccine status was not reliably recorded if patients received vaccines outside our healthcare system and it was not analyzed with respect to our outcomes. Testing for variant strain was rare and thus not available. Although variant strain could be obtained based on when infection occurred across the pandemic, there were often overlaps of multiple strains and thus, we did not analyze strains with respect to our outcomes. We analyzed COVID-19 severity with respective to outcome as it was good predictor. Immunity was not tested unless there was a clinical indication which was rare. COVID-19 treatments were heterogenous, included multiple combinations, changed across the pandemic, and were difficult to extract accurately; thus, it was difficult to compare systematically. The effects of these confounders on outcomes are complex and were difficult to assess. Future studies will need to include longer follow-ups and larger patient cohorts. As with any retrospective study, there could be other unintended patient selection biases and latent confounds.

## Conclusions

This study suggests that COVID-19 related hyperinflammatory syndrome confers a significantly higher risk for developing many new incident clinical disorders up to 3.5 years post infection. Identifying risks for developing new medical disorders in patients with COVID-19 related hyperinflammatory syndrome may encourage diligent follow-up of high-risk individuals.

## Electronic supplementary material

Below is the link to the electronic supplementary material.

Supplementary Material 1

## Data Availability

The datasets used are available from the corresponding author upon reasonable request.
