# Supplementary material for: Incidence and risk factors of new clinical disorders in patients with COVID-19 hyperinflammatory syndrome
Source: Sci Rep. 2025 Jun 6;15:19892. doi: 10.1038/s41598-025-04070-9 (PMC12144132; doi:10.1038/s41598-025-04070-9)
Supplement: Supplementary file 1 — Supplementary Material 1 [file 41598_2025_4070_MOESM1_ESM.pdf]

Supplemental Table 1. OMOP concept id's for common clinical disorders: hypertension, diabetes, cardiovascular diseases, chronic kidney disease, COPD and asthma. Cardiovascular diseases include congestive heart failure, coronary artery disease, and myocardial infarction.

**Hypertension**	
320128	Essential hypertension
**Diabetes**	
201254	Type 1 diabetes mellitus
201826	Type 2 diabetes mellitus
4193704	Type 2 diabetes mellitus without complication
443412	Type 1 diabetes mellitus without complication
**Cardiovascular Disease**	
319844	Acute ischemic heart disease
315286	Chronic ischemic heart disease
315296	Preinfarction syndrome
316427	Aneurysm of coronary vessels
321318	Angina pectoris
443563	Arteriosclerosis of coronary artery bypass graft
764123	Atherosclerosis of coronary artery without angina pectoris
4108215	Coronary thrombosis not resulting in myocardial infarction
4124683	Silent myocardial ischemia
4127089	Coronary artery spasm
36712779	Chronic total occlusion of coronary artery
36712982	Unstable angina co-occurrent and due to coronary arteriosclerosis
36712983	Angina co-occurrent and due to coronary arteriosclerosis
37115756	Dissection of coronary artery
37312532	Coronary arteriosclerosis in artery of transplanted heart
40481132	Arteriosclerosis of coronary artery bypass graft of transplanted heart
40481919	Coronary atherosclerosis
40482638	Arteriosclerosis of autologous vein coronary artery bypass graft
40482655	Arteriosclerosis of nonautologous coronary artery bypass graft
42537729	Aortocoronary bypass graft present
42537730	Coronary artery graft present
43021857	Arteriosclerosis of autologous arterial coronary artery bypass graft
4198141	Post infarct angina
432499	Mechanical complication due to coronary bypass graft
43020458	Mechanical breakdown of coronary artery bypass graft
319835	Congestive heart failure
439696	Hypertensive heart and renal disease with (congestive) heart failure
439846	Left heart failure
443580	Systolic heart failure
443587	Diastolic heart failure
444101	Hypertensive heart failure
4004279	High output heart failure
4014159	Chronic right-sided heart failure
4195785	Right heart failure secondary to left heart failure
4229440	Chronic congestive heart failure
4242669	Biventricular congestive heart failure

37309625	Acute on chronic right-sided congestive heart failure
40479192	Chronic systolic heart failure
40479576	Chronic diastolic heart failure
40480602	Acute on chronic systolic heart failure
40481043	Acute on chronic diastolic heart failure
44782719	Chronic combined systolic and diastolic heart failure
44782733	Acute on chronic combined systolic and diastolic heart failure
314666	Old myocardial infarction
438172	Atrial septal defect due to and following acute myocardial infarction
4108218	Subsequent myocardial infarction of inferior wall
4108219	Rupture of chordae tendinae due to and following acute myocardial infarction
4108220	Rupture of papillary muscle as current complication following acute myocardial infarction
4108677	Subsequent myocardial infarction of anterior wall
4108678	Hemopericardium due to and following acute myocardial infarction
4108679	Rupture of cardiac wall without hemopericardium as current complication following acute myocardial infarction
4108680	Thrombosis of atrium, auricular appendage, and ventricle due to and following acute myocardial infarction
4270024	Acute non-ST segment elevation myocardial infarction
4296653	Acute ST segment elevation myocardial infarction
4329847	Myocardial infarction
37309626	Myocardial infarction due to demand ischemia
37311078	Delayed postmyocardial infarction pericarditis
43020460	Acute ST segment elevation myocardial infarction involving left anterior descending coronary artery
45766114	Subsequent ST segment elevation myocardial infarction
45766241	Subsequent non-ST segment elevation myocardial infarction
46270162	Acute ST segment elevation myocardial infarction due to left coronary artery occlusion
46270163	Acute ST segment elevation myocardial infarction due to right coronary artery occlusion
**Chronic Kidney Disease**	
193782	End-stage renal disease
197921	Renal osteodystrophy
440302	Mechanical complication of peritoneal dialysis catheter
443597	Chronic kidney disease stage 3
443601	Chronic kidney disease stage 2
443611	Chronic kidney disease stage 5
443612	Chronic kidney disease stage 4
443614	Chronic kidney disease stage 1
443919	Hypertensive renal failure
4070976	Mechanical complication of dialysis catheter
4126451	Migration of peritoneal dialysis catheter
4127554	Failed renal transplant
4128369	Renal transplant rejection
43021418	Leakage of peritoneal dialysis catheter
43021985	Infection associated with peritoneal dialysis catheter
43531578	Chronic kidney disease due to type 2 diabetes mellitus
44782429	Chronic kidney disease due to hypertension
44782924	Misplacement of hemodialysis catheter
44784621	Hypertensive heart and chronic kidney disease
45763854	Chronic kidney disease stage 3A

45763855	Chronic kidney disease stage 3B
45768812	Anemia in chronic kidney disease
46271022	Chronic kidney disease
**COPD**	
255573	Chronic obstructive lung disease
255841	Chronic bronchitis
257004	Acute exacerbation of chronic obstructive airways disease
257905	Mucopurulent chronic bronchitis
261325	Pulmonary emphysema
261889	Simple chronic bronchitis
4110056	Chronic obstructive pulmonary disease with acute lower respiratory infection
4112826	Mixed simple and mucopurulent chronic bronchitis
4177944	Panacinar emphysema
4286497	Centriacinar emphysema
**Asthma**	
313236	Cough variant asthma
317009	Asthma
4138760	Exacerbation of intermittent asthma
4146581	Mild intermittent asthma
4279553	Eosinophilic asthma
37116845	Acute severe refractory exacerbation of asthma
45768910	Uncomplicated asthma
45768963	Uncomplicated mild persistent asthma
45768964	Uncomplicated moderate persistent asthma
45768965	Uncomplicated severe persistent asthma
45769350	Acute severe exacerbation of severe persistent asthma
45769351	Acute severe exacerbation of moderate persistent asthma
45769352	Acute severe exacerbation of mild persistent asthma
45769438	Acute severe exacerbation of asthma
45771045	Acute exacerbation of asthma
46270082	Acute exacerbation of mild persistent asthma
46273487	Acute exacerbation of moderate persistent asthma

Supplemental Table 2. Characteristics of patients who returned and didn't return.

Demographics	All returned (n = 8321)	Didn't return (n = 6014)	p-value
Age. Median (IQR)	65 (30)	68 (31)	<0.001
Female	4643 (55.80%)	3000 (49.88%)	<0.001
White	926 (11.13%)	819 (13.62%)	<0.001
Black	3010 (36.17%)	1857 (30.88%)	<0.001
Asian	262 (3.15%)	209 (3.48%)	0.30
Other	3783 (45.46%)	2654 (44.13%)	0.11
Hispanic	3762 (45.21%)	2477 (41.19%)	<0.001
Non-Hispanic	4225 (50.78%)	3011 (50.07%)	0.41
**Comorbidities**			
Hypertension	5468 (65.71%)	3313 (55.09%)	<0.001
Diabetes	3926 (47.18%)	2482 (41.27%)	<0.001
COPD	1344 (16.15%)	789 (13.12%)	<0.001
Asthma	2251 (27.05%)	1034 (17.19%)	<0.001
Cardiovascular Disease	3219 (38.69%)	2003 (33.31%)	<0.001
CKD	2685 (32.27%)	1573 (26.16%)	<0.001
Obesity	3895 (46.81%)	2251 (37.43%)	<0.001

Supplemental Table 3. Characteristics of COVID-19 hospitalized patients with cHIS and COVID-19 hospitalized patients with no cHIS. * indicates $p<0.05$, ** $p<0.01$, *** $p<0.001$ between cHIS and no cHIS. Laboratory values are the average readings within two weeks of index date.

	cHIS (n = 5459, 38.1%)	no cHIS (n = 8876, 61.9%)
**Demographics**		
Age, median (IQR)	70 (22) ***	63 (37)
Female %	2330 (42.68%) ***	5313 (59.86%)
White	655 (12.00%)	1090 (12.28%)
Black	1976 (36.20%) ***	2891 (32.57%)
Asian	188 (3.44%)	283 (3.19%)
Other	2640 (48.36%) ***	4612 (51.96%)
Hispanic	2192 (40.15%) ***	4047 (45.59%)
**Comorbidities**		
Hypertension	3664 (67.12%) ***	5117 (57.65%)
Diabetes	2868 (52.54%) ***	3540 (39.88%)
COPD	837 (15.33%)	1296 (14.60%)
Asthma	1026 (18.79%) ***	2259 (25.45%)
Cardiovascular Disease	2137 (39.15%) ***	2903 (32.71%)
CKD	2053 (37.61%) ***	2205 (24.84%)
Obesity or BMI > 30	2381 (43.62%)	3765 (42.42%)
Smoking	1802 (33.01%)	2806 (31.61%)
**Acute Outcomes**		
ICU/IMV	1288 (23.59%)***	447 (5.03%)
30 day mortality	1289 (23.61%)***	275 (3.1%)
In-hospital mortality	1227 (22.48%)***	238 (2.68%)
**Lab Values at Index (mean, sd)**		
Temperature (°F)	101.01 (1.67) ***	99.59 (1.66)
Ferritin (µg/L)	1649.09 (1616.58) ***	383.64 (580.92)
Neutrophil (k/uL)	11.60 (8.33) ***	7.08 (4.79)
Lymphocytes (k/uL)	1.86 (5.67) **	2.10 (2.74)
Hemoglobin (g/dL)	13.08 (2.27) ***	12.73 (2.03)
Platelets (k/uL)	335.05 (143.71) ***	281.91 (117.15)
D-dimer (mg/L)	6.34 (6.96) ***	1.62 (2.74)
LDH (U/L)	640.82 (958.41) ***	283.09 (251.93)
AST (U/L)	179.80 (735.05) ***	50.53 (217.47)
Triglyceride (mg/dL)	225.76 (249.95) ***	114.60 (72.76)
CRP (mg/L)	173.83 (118.26) ***	53.26 (62.14)
WBC (k/uL)	14.22 (11.72) ***	9.70 (6.99)
Creatinine (mg/dL)	2.95 (3.93) ***	1.54 (2.06)

Supplemental Table 4. Univariable hazard ratios for developing new hypertension, diabetes, cardiovascular diseases, chronic kidney disease, COPD and asthma. * indicates $p<0.05$, ** $p<0.01$, *** $p<0.001$ between cHIS and no cHIS.

	Hypertension	Diabetes	COPD	Asthma	Cardiovascular Disease	CKD	Obesity
cHIS	2.01 (1.66,2.43)***	1.33 (1.05,1.69)*	1.35 (1.06,1.71)*	0.90 (0.67,1.20)	1.75 (1.48,2.07)***	1.63 (1.33,2.00)***	1.48 (1.21,1.81)***
Female	0.67 (0.56,0.81)***	0.86 (0.69,1.09)	0.86 (0.68,1.09)	1.18 (0.90,1.54)	0.77 (0.65,0.91)***	0.80 (0.65,0.98)*	1.30 (1.07,1.58)*
Age	1.03 (1.02,1.03)***	1.02 (1.02,1.03)***	1.04 (1.03,1.04)***	0.99 (0.98,0.99)***	1.04 (1.03,1.04)***	1.04 (1.03,1.04)***	1.00 (0.99,1.00)
Hispanic	0.85 (0.7,1.03)	0.92 (0.73,1.16)	0.78 (0.61,0.99)*	1.02 (0.78,1.34)	0.85 (0.72,1.01)	0.73 (0.60,0.90)***	1.31 (1.08,1.60)*
Black	1.21 (0.99,1.46)	1.10 (0.87,1.39)	1.22 (0.96,1.55)	1.13 (0.86,1.49)	1.11 (0.94,1.32)	1.67 (1.36,2.04)***	0.86 (0.70,1.07)
Hypertension	na	2.46 (1.93,3.15)***	3.14 (2.29,4.31)***	0.95 (0.72,1.26)	2.97 (2.44,3.61)***	4.10 (3.12,5.38)***	0.92 (0.76,1.13)
Diabetes	2.58 (2.13,3.13)***	na	2.01 (1.58,2.55)***	0.86 (0.65,1.13)	2.38 (2.01,2.81)***	2.64 (2.16,3.23)***	1.20 (0.98,1.46)
COPD	1.39 (1.01,1.92)*	1.45 (1.07,1.96)*	na	2.71 (1.98,3.71)***	2.08 (1.66,2.61)***	1.69 (1.31,2.18)***	0.72 (0.53,0.97)*
Asthma	0.74 (0.58,0.93)*	1.20 (0.93,1.54)	1.85 (1.44,2.36)***	na	1.03 (0.86,1.25)	0.94 (0.75,1.18)	0.94 (0.74,1.20)
Cardiovascular Disease	2.50 (2.03,3.07)***	2.58 (2.05,3.24)***	2.48 (1.96,3.14)***	1.41 (1.08,1.86)*	na	2.44 (2.00,2.98)***	0.80 (0.65,0.98)*
CKD	3.05 (2.49,3.73)***	1.92 (1.49,2.48)***	2.04 (1.61,2.58)***	0.99 (0.74,1.32)	2.38 (2.00,2.83)***	na	0.78 (0.62,0.97)*
Smoking	1.33 (1.08,1.64)*	1.22 (0.96,1.55)	2.22 (1.76,2.81)***	1.54 (1.17,2.02)***	1.42 (1.19,1.69)***	1.16 (0.94,1.43)	0.85 (0.69,1.05)
Obesity	1.27 (1.05,1.53)*	1.40 (1.12,1.76)***	1.18 (0.94,1.50)	1.08 (0.82,1.41)	1.14 (0.97,1.35)	1.07 (0.88,1.31)	na

Supplemental Table 5. cHIS prediction of COVID-19 critical illness (ICU and IMV) and in-hospital mortality. This is an additional validation to determine whether cHIS is valid predictor of acute COVID-19 severity.

	Critical illness			Mortality		
	AUC	Sensitivity	Specificity	AUC	Sensitivity	Specificity
cHIS > 2	0.74	0.99	0.48	0.69	0.91	0.48
cHIS > 3	0.84	0.99	0.68	0.75	0.83	0.67
